# Design of network English autonomous learning education system based on human-computer interaction

**DOI:** 10.3389/fpsyg.2022.989884

**Published:** 2022-09-21

**Authors:** Xin Wang, Simon Smith

**Affiliations:** ^1^College of Foreign Studies, Nanjing Agricultural University, Nanjing, China; ^2^School of Humanities, Coventry University, Coventry, United Kingdom

**Keywords:** human-computer interaction (HCI), autonomous English learning system (AELS), Optimized Deep Learning Network (ODNN), independent learning, education system

## Abstract

The continuous development of Human-Computer Interaction (HCI) and information technologies impact the digital learning environment. The network and multimedia technologies change the Autonomous Learning System (ALS) structure. The learning process uses several techniques; however, the interactive function requires continuous improvement to enhance autonomous learning. Therefore, Optimized Deep Learning Network (ODNN) is introduced to build the Autonomous English Learning System (AELS) in this work. The ODNN system uses the learning and activation functions that derive the student’s learning capabilities and gives the proper training to the student. The HCI-based created autonomous learning process provides the guidelines to the student for making independent learning. The ALS improves the student’s learning ability and skills compared to classroom-based learning. The discussed ODNN-based AELS system effectiveness is evaluated using the Japanese-English Bilingual Corpus with a set of assessment questionaries. Then the HCI-based autonomous English learning is a quantitative analysis with the classroom-based learning. The discussed system is implemented using the Python tool, in which the AELS system ensures 98.51% learning efficiency compared to classroom learning.

## Introduction

Nowadays Human-Computer Interaction (HCI) system (Adem et al., 2022) plays a vital role in different multidisciplinary fields such as healthcare, education, sports etc. The HCI system uses computerized technologies for making the interaction between the user (human) and computers (Al Mahdi et al., 2019). The HCI process supports the product’s functionality, usefulness, safety and success. Therefore, the HCI-based applications are widely utilized by users to achieve the target in a short period. However, the HCI system should concentrate on skill-based system development for people who do not complete the proper training. Significantly, the HCI environment works for the learning system (Torres-Carrión et al., 2018; Dingli and Caruana Montaldo, 2019) because it requires Web-based or online learning. The learning platform represents the Technology Enhanced Learning (TEL) (Mogoş et al., 2018), empowered by various digital technologies. Traditionally, the learning process is performed according to human-human interaction only (Stefanidi et al., 2022). However, the HCI development causes the establishment of the link between the user interface and the computer. The association between the computer and the user pushes to access explicit and implicit information *via* the machine (del Carmen Rodríguez-Hernández and Ilarri, 2021; Mukherjee et al., 2022). Therefore, developing the HCI systems, methods and theories leads to developing the autonomous English Language learning systems. Among the various technologies, the highly influenced approach is Neural Networks (NN), which are used to create the effective Intelligent Education System (IES) (Wang F. et al., 2020).

In IES, students must continuously update their skills and knowledge to survive in this globalized society (O’Dowd, 2020). The students don’t receive enough new information in school learning. The students and people should be aware of the independent learning technologies to meet the education goal (Lv et al., 2017; Grange and Barki, 2020). Therefore, this research work uses the HCI technologies to improve students’ English learning (Hu et al., 2021; Wu, 2021). The effective utilization of the NN with the HCI process gives better results in an Autonomous English Learning System (AELS) (Arevalo, 2021). In addition, the education system continuously performs education reform, requiring the independent English learning process to meet the requirements (Gibson et al., 2020). Then the HCI-based learning process has three stages: searching, self-study program and self-learning. In the searching process, the student uses the English keywords analyzed on the Internet to get the knowledge. Then the self-study program has to be implemented because the English learner has limited materials.

Finally, self-learning strategies are identified for improving their learning behavior. During this process, users create their self-learning strategies, goals and learning process to enhance their learning skills (Yang, 2018). The autonomous learning process works in two processes: staffs continuously take their training according to the pre-prepared videos and quizzes. According to the trained information, AELS is deployed (Merritt, 2021). Further, staff are clearing their doubts and encouraging students online. Even though autonomous learning provides an effective learning platform, it has a few challenges, such as difficulty in identifying the starting point of education, lack of motivation, time and interest to gain knowledge *via* the Internet source. Therefore, several researchers are interested in creating the HCI system for developing the autonomous learning system (ALS). However, the interactive functions are still weak to ensure effective results. In this educational system, pupils’ ability to learn on their own is promoted *via* the use of ongoing assessments of a variety of competencies. Evaluation of competency is the process of gathering evidence on the accomplishment of an individual’s brilliant practice to create a judgment about his/her abilities with respect to a professional profile. When making a choice, an assessment is a process of gathering information, processing it, interpreting it, and employing it.

Then the AELS issues are overcome by applying the HCI with the machine learning techniques. Optimized Deep Learning Network is applied to investigate student learning patterns. During the learning process, the deep neural model is updated using the bee colony algorithm to reduce the deviation between the actual learning and independent learning outputs. For an argumentative design, the interplay between cognitive psychology and HCI must be considered. Evaluation of the student’s comprehension of course content and analysis of difficulty levels for test questions are the app’s primary functions. This research developed a control experiment to test the influence of HCI teaching on students’ English proficiency and to examine students’ performance and instructors’ sentiments and their views on HCI learning. Computing, cognitive psychology, and ergonomics are major influences on HCI design. User interfaces, as they are perceived and controlled by humans, are its primary focus. Using this method, we may see humans and machines as two interdependent systems that exchange information in real-time. Then, a continuous testing procedure is implemented to evaluate the student learning strategies. The system’s effectiveness was assessed using performance analysis and implemented using Python.

### The main contribution of this study

(i) Student learning and activation functions are derived from the ODNN system, which provides the student with appropriate instruction.
(ii) HCI-based autonomous learning offers students a framework for self-directed study.
(iii) Thus, a quantifiable comparison may be made between HCI and conventional teaching methods.

Then the rest of the paper is organized as follows: Section “Related works” analyses the various researcher’s opinions regarding the HCI-based English learning system. Section “Human-computer interaction-based autonomous English learning system” discusses the working process of the HCI-based ODNN approach related to AELS and the system’s effectiveness evaluated in section “Results and discussions.” The conclusion is described in section “Conclusion.”

## Related works

Various researchers’ work, ideas, frameworks and interactive technologies are analyzed in this section to improve the autonomous learning education system. Pikhart (2021) analyzed foreign language learning applications of HCI in the mobile learning process. The analysis uses the University of Hradec student’s or user information details for the qualitative analysis. During the investigation, 18 students are selected according to their foreign language application usage. The collected details investigated in terms of the qualitative way users require additional Artificial Intelligence (AI) efforts to improve the learning process. Although the applications were relatively new, the study’s results clearly reveal that users’ primary issue was the absence of AI use and the apps’ clunky design.

Li (2022) developed HCI based intelligent English learning systems. The main intention of this study is to reduce the difficulties in the interactive function utilized in the learning process. The system uses the assisted learning and learning function to understand English content. According to the learning process, an intelligent learning system is created, and the pronunciation score value is utilized to evaluate the system. The successful verification process minimizes the English learning difficulties and improves students’ understanding. It’s feasible to address the issue of restricted learning time more effectively with the help of ICT tools. To make up for the limitations of English classroom teaching and successfully tackle the practical challenges experienced by students in English learning, it is intended this approach would be used.

Tian (2020) investigated the computer network environment for improving English classroom teaching in college. The research uses network and computer information technologies to improve English learning in college. The effective utilization of these network technologies supports the college teaching reforms and also manages the teaching quality and efficiency. There are several advantages to using multimedia teaching tools, such as providing a wide range of materials and laying a solid foundation, as well as allowing for easy and sophisticated technological development.

Kuo et al. (2019) introduced the Interdisciplinary Project-Based Learning (IPBL) approach to motivating the student learning process in Science, Technology, Environment and Mathematics (STEM) environment. During the analysis, 45 students are selected along with their STEM courses to identify their learning knowledge and skills. The study uses the Motivated Strategies related Learning Questionnaires (MSLQ) to evaluate student learning skills. The MSLQ questionaries are more valuable for understanding the overall creativity, knowledge, and talent during the learning process.

Sfenrianto et al. (2018) analyzed adaptive learning systems according to the knowledge level of student English learning. This system intends to examine the English learner’s proficiency level. The learning system gives the pre-test for every learner to understand the student learning stage. According to the test value, learners are categorized as elementary, intermediate and advanced. Then the adaptive system gives the guidelines and materials to the student to improve their English proficiency.

Aizawa and Yoshimura (2021) created an English learning system using the Near Field Communication (NFC) tag. The research analysis uses the Raspberry Pi and NFC readers to analyze the learning efficiency. Initially, NFC tags are investigated because they relate to the words and illustration. Then tag value is computed related to the English terms the readers hold. According to the NFC, readers having pair answers are displayed, which helps to improve the student learning process. In addition, Information and Communication Technologies (ICT) are incorporated to develop an effective English learning system.

Zhao (2022) designed an English auxiliary teaching system by applying Artificial Neural Networks. The systems aim to create the English Assistant Instruction System (EAIS) for China students. The neural network uses the student academic performance data to analyze the English learning level. The neural network computes the average monthly test score to determine the learning efficiency. The successful utilization of learning function in neural networks improves the overall auxiliary system learning rate from 81.5 to 90.11%.

Hou (2021) developed a Support Vector Machine (SVM) and Decision Tree (DT) based online teaching system quality evaluation. The intelligent method uses the adaptive learning rate and gradient descent methods for maximizing the convergence rate while performing quality analysis. Complex data relationship is computed during the investigation to minimize the difficulties in high-dimensional data analysis. According to the data relationship, the student learning effectiveness is evaluated and compared with the existing methods.

Khamparia and Pandey (2018) developed an E-learning system using the Support Vector Machine (SVM) and Principle Component Analysis (PCA) based learning features. The author investigates the student’s eight learning attributes such as Learning style (L), Anxiety (A), previous sem grade (PSG), Cognitive style (C), Personality (P), Study Level (SL), Prior Knowledge of student (SPK), and Motivation (M). These attributes are collected from National Centre Biotechnical Information (NCBI) E-learning database. The collected information is processed by least square SVM and Feed Forward Neural Networks (FFNN), which helps identify the student’s learning level effectively. According to the existing researcher’s opinion, the AELS is developed by applying the various ICT approaches. However, the English Language requires continuous effort to improve the learner’s efficiency. In addition, the ALS requires an interactive process to enhance the overall learning rate. Therefore, in this work, HCI system is incorporated into the English Learning process to achieve the learner’s efficiency. In this work, Optimized Deep Learning Network (ODNN) approach is integrated with the HCI system to identify student English learning abilities. Then the detailed working process of HCI with ODNN-based autonomous English Learning process is discussed below.

## Human-computer interaction-based autonomous English learning system

The research intends to construct the HCI-based AELS. The AELS system is developed by incorporating the Optimized Deep Learning Networks (ODNN). The constructed intelligent learning system is used to understand the audience’s mindset and strategies for improving the learning rate. The ALS developed in terms of two stages: academic-related (medical research, agricultural engineering, mechanical engineering, etc.) and basic knowledge-related learning (Ding et al., 2019; Wang X. et al., 2020). Initially, the interaction system uses the basic knowledge of English which is utilized to understand the prepare the audience to use the system. In addition, the students learning capabilities, interests, learning styles and characteristics are investigated to improve the teaching methods. Therefore, the AELS system requires the most relevant learning and resources to enhance learner efficiency. The HCI-based AELS system should follow the teaching level and technical knowledge strategies to facilitate in-depth English language knowledge.

Generally, multimedia classrooms utilize the campus Internet while accessing the learning information. The campus network resources act as the bridge between the students and staff, and the multimedia-based learning process improves students’ interests. Then the student learning capabilities are continuously enhanced by performing the educational reforms and the respective high-quality resources shared *via* the network. It is possible to employ technology to assist and enhance one’s ability to learn a foreign language. Teaching foreign languages has never been easier than it is now, in large part to the flexibility afforded by technological advances. Teachers are increasingly relying on technology to assist their students in learning a new language. However, most students lack knowledge of English, which ultimately affects the student’s learning progress. Generally, students face difficulties in grammar; therefore, the HCI-interaction system should concentrate on essential learning skills such as sentence structure, grammar and vocabulary. Especially in the foreign language department, students face vocabulary difficulties, sentence structure formation and grammar issues. Therefore, tutors should address these challenges while teaching English because the problems affect the students learning performance. Accordingly, a computer interaction system should be developed to cover the curriculum reform that covers several leading such as writing, reading, listening and speaking. These modules should cover the grammatical phase because it is the backbone of the autonomous English learning process.

### Human-computer interaction with ODNN-based autonomous English learning system

This section discusses the HCI Optimized Deep Learning Networks (ODNN) approach to AELSs. The system consists of four modules: maintenance according to knowledge, diagnosis practices, strengthening of knowledge points and improvements. It’s this participatory learning on the web that teaches users the right technique to model data. Simply said, it’s easy to use and very efficient. In this regard, the emphasis of this work is on Human-Computer Interface (HCI) design concepts and execution (HCI). HCI design concepts and principles are laid forth, and an interactive e-Learning application’s HCI design is evaluated.

**FIGURE 1:**
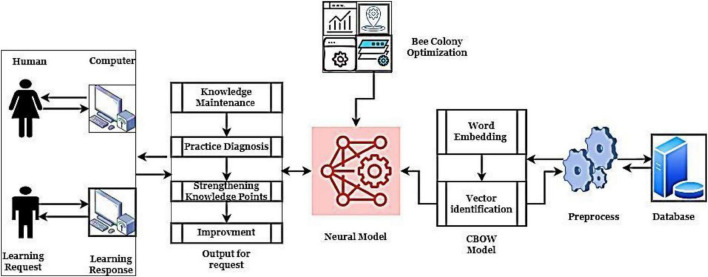
HCI with ODNN-based AELS structure.

These four modules are critical points for the HCI with an autonomous learning process which works according to the machine learning algorithms. The collected English dataset has been investigated using Natural Language Processing (NLP) to perform the preprocessing, which improves the overall AELS system performance. This work uses optimized deep learning networks to examine students’ learning behavior. The network examines the relationship between the students learning process with their performance. The effective utilization of the network activation and learning function reduces the system complexity. During the computation, the network uses fault tolerance, parallelism, numerous approximation, self-learning and hardware properties to improve the AELS performance. Then the HCI with ODNN-based AELS system working process is illustrated in Figure 1.

Figure 1 illustrates the working structure of HCI with the ODNN-based AELS process. Here, student request has been processed by investigating the database. Regarding the HCI and cognitonics elements of the user interface, this study is concerned. A high-quality user interface can only be achieved by doing usability tests using HCI-specific approaches. According to the request, the computer analyses the response and the results are mapped with the request. The learner’s answers are analyzed, and a vector model is derived to predict the matching with the template with the database. During this process, bee colony optimization (Alaidi et al., 2021) updates the network parameters. The network provides the output, and according to the output, knowledge points are investigated successfully. There’s a chance to learn about Information and Communication Technology (ICT). In this article, we provide the results of our investigation into designing an English learning platform.

#### Maintenance according to knowledge

The first module of the AELS is knowledge maintenance which only the English experts do. The above Figure 1 constructed neural network has input, hidden, and output layers along with the bee colony-based updated network parameters. These optimized parameters played a vital role in network design to improve learning efficiency. The updating procedure utilizes the learning samples for identifying the deviation between the student learned and label inputs. The knowledge maintenance process is used to understand the student learning characteristics and network functions. The English experts arrange the input samples continuously fed into the neural networks to create the knowledge base for improving the learning patterns. The student’s outputs are compared with the learning patterns to identify the deviations and optimization problems. Suppose the created system fails to adopt the expert’s knowledge; they are trying to change, modify and delete the samples from the knowledge dataset to improve the network performance.

#### Diagnosis practices

The following important module is diagnosis practices, in which the system continuously learns the samples from expert knowledge. The system learns the several knowledge points in the example question banks. Then the questions are given to the students, and the student’s answers are compared with the training set, which helps to make the self-diagnosis. According to the self-diagnosis process, students learning ability is understood, and their learning level has to be identified effectively.

#### Strengthening of knowledge points

The neural network should consider the different knowledge points to ensure effective AELS. Therefore, the learning points are classified as various points such as untested knowledge points, good knowledge points and low knowledge points. These knowledge points are utilized for examining the students learning capabilities. Students can select the respective modules to make their learning and testing process. After the learning process, self-diagnosis is taken to evaluate their learning performance.

#### Improvements

The last module of the AELS is practice improvement, which is done between students and skilled users. The students learning ability is checked continuously, and the results are matched with the database. During the analysis, student ability is analyzed at a specific time to determine their learning efficiency. The improvements are examined in terms of historical practices and knowledge point exercises. The student results are consolidating their memory and forgetting category. Finally, the AELS system combines the students’ knowledge and improvement points in English learning.

These four modules require a database to evaluate the student learning efficiency. The dataset consists of student personal information (user name, user category, and password) and a user static knowledge table (the knowledge table maintained by experts). The stationary knowledge tables consist of a set of English vocabulary details and grammatical information used to identify the user effectiveness. The static knowledge table has knowledge points and question banks (domain experts test questions, options, questions, and answers). In addition, rule knowledge points (topic number, certainty, knowledge point number) are maintained to balance the learning efficiency. The constructed knowledge information is processed by applying the Optimized Deep Learning Networks (ODNN). The neural network process the collected question banks (text) along with the Natural Language Processing (NLP). The constructed HCI system-based AELS helps understand the user requirements, and the learning process is established accordingly. The NLP techniques investigate the student’s answers in terms of examining sentence and word count, Parts of Speech (PoS) Tag, spelling mistakes and frequent occurred words. The sentence and word count use the “text mining” library tool for investigating the number of words in the sentence. The library has 276 stop words in English; according to these words, sentences and words are counted continuously. Then Parts-of-Speech (PoS) tag details are extracted from the student’s answer. Here Natural Language Tool Kit (NLTK) is utilized to extract the PoS tags from the given input English sentence. Then the student’s structural and textural features are extracted to determine the frequency of occurrence of words. Consider the student’s answer; it has a set of features and weight values that are represented as {*f*_1_,*f*_2_,…..*f*_*n*_} and the {*w*_1_,*w*_2_,…..*w*_*n*_}. From the set of features, the Term Frequency and Inverse Term Frequency (TF-ITF) value is estimated for identifying the student learning performance. Then the Term Frequency (TF) is calculated using Eq. 1.


(1) $$T\,F_{i,k}=n_{i,k}$$

In the English language, words are presented in various positions and have different lengths; hence, the TF value should be standardized using Eq. 2.


(2) $$T\,F_{i,k}=\frac{n_{i,k}}{\sum_{m}n_{m,k}}$$

It is possible to identify the “signature” of a writer’s cultural level, slang or technical words, and other literary qualities using frequency analysis. It is feasible to infer a person’s overall vocabulary from the number of words used in a particular document. With the help of the TF value, the Inverse Document Frequency (IDF) has been computed using Eq. (3).


(3) $$I\,D\,F_{i,k}=l\,o\,g\,\frac{\left|D\right|}{\left|\left\{d:\,t_{i,k}\in d\right\}\right|}$$

From the computed term frequency value *t*_*i*,*k*_, term m-composition has been computed using Eq. (4).


(4) $$IDF_{i,k}=log\left(M|m_{i,k}+\propto \right)$$

**FIGURE 2:**
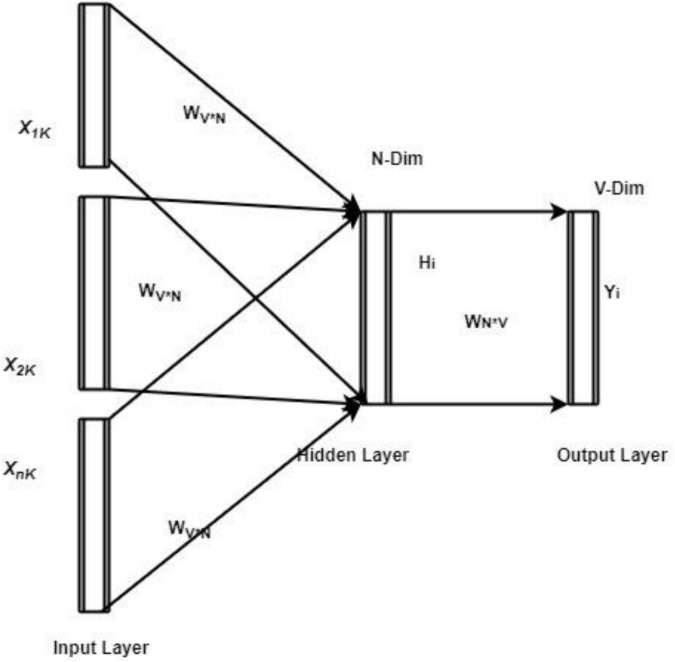
CBOW-neural model structure.

In Eq. 4, ∝ it is represented as the constant, and its value is 0.01; the weight value is computed as *w*_*i*,*k*_ = *TF*_*i*,*k*_ + *IDF*_*i*,*k*_. Then sentence length, unique terms and characteristics are extracted from student answers. After applying the NLP process, word embedding is performed to extract the vocabulary from the student document. The embedding process helps get the syntactic and semantic similarity values and the relationship between the words. The Word2Vec constructs the embeddings from the sentence using the Common Bag of Words (CBOW). The CBOW model derives the context values of words, and the related terms are predicted during the training and testing process. For every input, CBOW uses the neural network layers that have been analyzing each word, and the respective context words are predicted using the database. During this process, the input word hot encoding value is utilized to estimate the output or target word. The input word is one of the encoded vectors, and the one-hot encoded vector has V in size. These input vectors are processed using the hidden layers with N neurons, and the computed output value is estimated with the same V size, which is done using the softmax function. The network has the weight value between the input *x* to the hidden layer (V*N−*dimensionalmatrix*) and the hidden layer to the output layer (N*Vdimensionalmatrix). Then the structure of the CBOW-related neural network is illustrated in Figure 2.

This work applies an optimized deep neural model to manage students’ English learning process. The effective utilization of the neural word embedding process retains the system’s robustness and effectiveness and achieves good performance. The ODNN model must be utilized to evaluate the student’s answer; their performance evaluates the learner’s efficiency. A deep learning network processes the extracted input features with several layers such as input, hidden and output layers; each layer performs a specific function to attain the output. The extracted inputs are investigated in the convolution layer that uses the filters to reduce the data size. Then the max-pooling process is applied to minimize the data size and eliminate the over-fitting issue. The outputs are obtained using the softmax activation function, and the computed output is compared with the training set. The deviation between the output values is calculated to update the network parameter. The updating procedure is performed continuously to improve the overall AELS performance. This work uses the bee colony optimized algorithm to select the suitable network parameters. The bee colony optimization algorithm selects the parameter based on the food searching process. It has bee, and a set of food sources are the central concept while selecting the features. During the search process, nectar values are used to choose the best food source to resolve the optimization problem. The algorithm has scouted, employed and onlooker bees that highly influence the food searching. The employee bee checks the parameters, and the computed values are transferred to the scouts while the searching space does not have the food resource. The searching process is initialized by using Eq. 5.


(5) $$x_{i}^{j}=x_{m\,i\,n}^{j}+r\,a\,n\,d\,\left(0.1\right)\,\left(x_{m\,a\,x}^{j}-x_{m\,i\,n}^{j}\right)$$

In Eq. (5), solutions are predicted from ith iteration in search space, the search space having the number of solutions which are belonging to the neural model weight value, and the answer is computed from the minimum $x_{m\,i\,n}^{j}$ and maximum $x_{m\,a\,x}^{j}$ of the solution in search space. After initializing the search space, the employee bee searching process is performed that is computed using Eq. (6).


(6) $$v_{i}^{j}=x_{i}^{j}+\varphi_{i}^{j}\,\left(x_{i}^{j}-x_{k}^{j}\right)$$

In Eq. 6, k is the random index value taken from 1 to *n* (number of solutions), a random decimal value $\varphi_{i}^{j}$ is from −1 to 1, and the new searched food is defined as the $v_{i}^{j}$ that is computed from *x*_*i*_. The computed food resources are compared with the new resources to identify the best food resources. The computed solution is transferred to the onlooker bee that identifies the optimized solution from the solution set. The new solutions are selected based on the nectar value; if the solution has a maximum value, it has been added to the list, and previous solutions are eliminated. Then the new solutions are computed using Eq. (7).


(7) $$p_{i}=\frac{f\,i\,t\,\left(x_{i}\right)}{\sum_{n=1}^{B\,N}f\,i\,t\,\left(x_{n}\right)}$$

According to Eq. 7, the optimal solutions are identified, and the local optimal solution issue is resolved using the scouting stage activity. After identifying the solutions, the optimal new solutions are obtained by performing the mutual learning that is defined in Eq. (8).


(8) $$v_{i}^{j}=\left\{ \begin{array}{c} x_{i}^{j}+\varphi_{i}^{j}\,\left(x_{K}^{j}-x_{I}^{j}\right),F\,i\,t_{i}<F\,i\,t_{k}\\ x_{K}^{j}+\varphi_{i}^{j}\,\left(x_{i}^{j}-x_{k}^{j}\right)\;F\,i\,t_{i}\geq F\,i\,t_{k}\; \end{array}\right.$$

**FIGURE 3:**
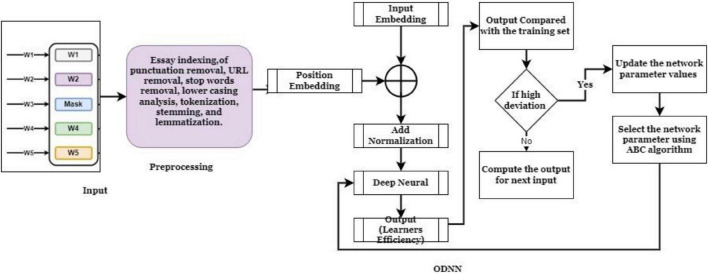
Working process of ODNN.

In Eq. 8, current and neighboring food source fitness values are represented as the *Fit*_*i*_ and *Fit*_*k*_. A Uniform random number is defined as $\varphi_{i}^{j}$. Which has a value from 0 to F in which F is denoted as the non-negative constant value or mutual learning factor. The computed new solution-based neural model weight parameters are selected for updating the network parameter. Effective learning and solution updating strategies effectively minimize the exploration and exploitation problem. Network parameters are updated depending on the above optimization process, and the respective function is illustrated in Figure 3.

According to Figure 3, the students/learner’s English efficiency is computed, and the respective actions are taken. If the students obtained high matching values with the training set, they require minimal effort to improve their English learning process. If the student attains less matching value, then the AELS process gives the needed knowledge points to them. There are two primary paths for future development. One focuses on the correct examination of the underlying data, while the other focuses on additional HCI and cognitonics study of the generated tool. Then the detailed steps are included in Table 1.

**TABLE 1:** Working process of HCI with ODNN based AELS.

Step 1: Collect the English sentence from the experts and form the database
Step 2: Analyzing the student inputs and unwanted information like punctuation, stop words and lower case details are removed.
Step 3: Applying the word embedding process to extracting the vectors and position details
Step 4: Evaluate the vectors using the neural model to identify and verify the student answer.
Step 5: Compare the output with the database template
Step 6: if the variation is high then the network parameter needs to be updated using bee colony algorithm
Step 7: Select the network parameter according to the solution probability value
Step 8: Estimate the students learning efficiency value.
Step 9: Repeat the process.

Therefore, HCI based autonomous English learning process provides the proper guidelines to learners. Then the effectiveness of the system is evaluated using experimental results and discussions.

## Results and discussions

This section discusses the effectiveness of the introduced HCI with Optimized Deep Learning Network (ODNN) based AELS. This process uses the Japanese-English Bilingual Corpus^1^ (Kaggle, 2017) with a set of questionaries. The dataset consists of around 500,000 pairs of manually translated sentences, which is the corpus’s enormous scale. The collected sentences were utilized for training the AELS to improve their learning performance. The dataset covers various topics such as Japanese religion, history and culture. In addition, the dataset has Kyoto Lexicon, which is developed by deriving the Japanese-English word pair. Here, the Japanese students face technical difficulties, not essential knowledge. Most of the AELS assist the students in understanding the comprehensive applications instead of concentrating on basic English knowledge. The learners utilize the constructed HCI system to store massive data to enhance the complete analysis. However, the developed system provides comprehensive expertise and focuses on basic knowledge to improve the autonomous English learning process. The developed HCI-based AELS process is utilized *via* the applications because the APP has a recording function and microphone that helps to understand the articles and is used to practice the pronunciation. Moreover, the application constructs with the specific theme-related ideas used to learn English autonomously. The created AELS process is designed with two phases: repeating what they heard and writing down the heard content. During the learning process, the practice section gradually increases according to the speaking speed and length of the sentence. The constructed ODNN-based learning process was evaluated using the following metrics


(9) $$A\,c\,c\,u\,r\,a\,c\,y=\frac{T\,r\,u\,e\,P\,o\,s\,i\,t\,i\,v\,e+T\,r\,u\,e\,N\,e\,g\,a\,t\,i\,v\,e}{T\,r\,u\,e\,p\,o\,s\,i\,t\,i\,v\,e+T\,r\,u\,e\,N\,e\,g\,a\,t\,i\,v\,e+F\,a\,l\,s\,e\,P\,o\,s\,i\,t\,i\,v\,e+F\,a\,l\,s\,e\,N\,e\,g\,a\,t\,i\,v\,e}$$

(10) $$R\,e\,c\,a\,l\,l=\frac{T\,r\,u\,e\,P\,o\,s\,i\,t\,i\,v\,e}{T\,r\,u\,e\,P\,o\,s\,i\,t\,i\,v\,e+F\,a\,l\,s\,e\,N\,e\,g\,a\,t\,i\,v\,e}$$

(11) $$P\,r\,e\,c\,i\,s\,i\,o\,n=\frac{T\,r\,u\,e\,P\,o\,s\,i\,t\,i\,v\,e}{T\,r\,u\,e\,P\,o\,s\,i\,t\,i\,v\,e+F\,a\,l\,s\,e\,P\,o\,s\,i\,t\,i\,v\,e}$$

(12) M⁢S⁢E=A⁢c⁢t⁢u⁢a⁢l⁢V⁢a⁢l⁢u⁢e-C⁢o⁢m⁢p⁢u⁢t⁢e⁢d⁢V⁢a⁢l⁢u⁢e

(13) $$L\,e\,a\,r\,n\,i\,n\,g\,E\,f\,f\,i\,c\,i\,e\,n\,c\,y=\frac{T\,N*T\,P-F\,P*F\,N}{\sqrt{\left(T\,N_{F\,N}\right)\,\left(F\,P+T\,P\right)\,\left(T\,N+F\,P\right)\,\left(F\,N+T\,P\right)}}$$

(14) $$L\,e\,a\,r\,n\,i\,n\,g\,R\,a\,t\,e=\frac{2\,T\,r\,u\,e\,P\,o\,s\,i\,t\,i\,v\,e}{2\,T\,r\,u\,e\,P\,o\,s\,i\,t\,i\,v\,e+F\,a\,l\,s\,e\,N\,e\,g\,a\,t\,i\,v\,e+F\,a\,l\,s\,e\,P\,o\,s\,i\,t\,i\,v\,e}$$

In Eqs 9–11, True Positive (TP) is defined as a correctly identified student answer, True Negative (TN) is defined as a model that effectively identifies the wrong answer, False Positive (FP) is defined as falsely rejecting the null value, and False Negative (FN) is defined as wrongly identifies the student’s answers.

**FIGURE 4:**
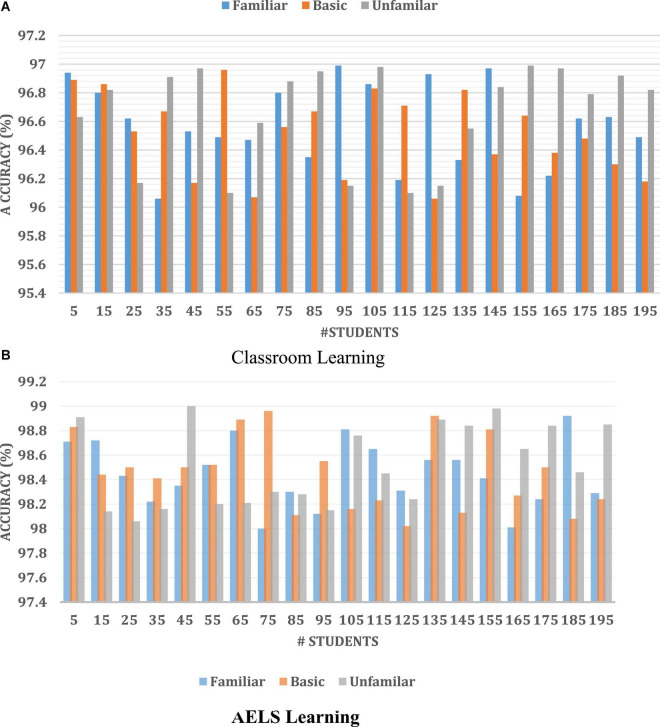
Student textbook and AELS App-based learning process efficiency.

Then the HCI with ODNN-based AELS system learning efficiency is compared with the traditional classroom environment. During the analysis, around 195 students learning efficiency is compared, and the results are illustrated in Figure 4.

**FIGURE 5:**
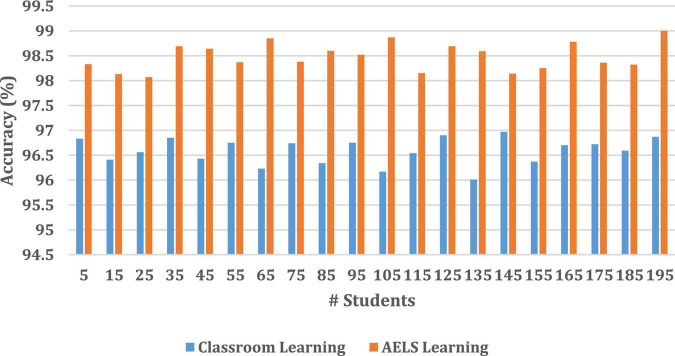
Accuracy analysis **(A)** classroom learning **(B)** AELS learning.

Figure 5 clearly states that the introduced AELS learning system ensures high accuracy while students do their learning process. The AELS system was created with vast data covering almost every comprehensive and basic English knowledge information. Therefore, the traditional classroom-based learning process consumes high time to understand the basic concept. According to Figure 5, the independent learning process investigated for 195 students, the textbook-based learning process improves only 1.04% of the learning process. The AELS-based learning process increases learning by up to 3.15%. The analysis clearly states that students are interested in learning English *via* the AELS process. The HCI-based AELS system utilizes the ODNN approach to investigating the student knowledge points. According to the student’s query, the constructed AELS system reacted by analyzing the database information. Here, the collected dataset information is divided into two testing (30%) and a training set (70%). Once the dataset is collected, it is processed by applying the NLP techniques to eliminate the irrelevant information from the student’s answer. Then the efficiency of the introduced ODNN approach is evaluated in three phases such as familiar, basic, and unfamiliar. Then the HCI- with ODNN-based AELS system effectiveness is illustrated in Figure 4.

**FIGURE 6:**
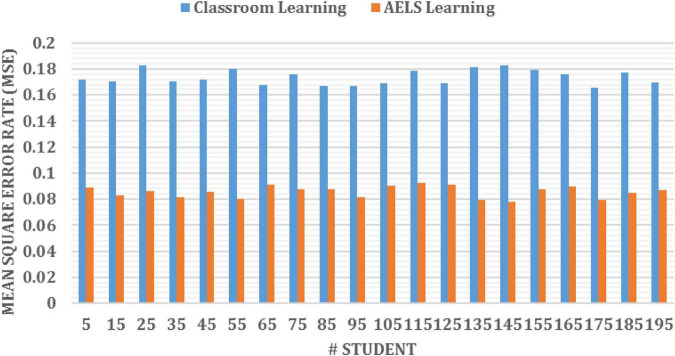
Error rate analysis.

Figure 4 clearly shows the effectiveness of the introduced ODNN approach-based AELS. During the analysis, the system uses the basic, familiar and unfamiliar concepts to evaluate the student’s English learning efficiency. The deep neural network utilizes multiple layers for processing the student inputs. Here the set of language sentences is collected, and respective embedding vectors are derived that are more useful for processing the learner’s request. The students are investigated by conducting a test in which sample questionnaires are asked to evaluate their performance. The performance analysis is done on three levels: basic, familiar and unfamiliar. The AELS system prepares the answers for respective questions stored in the training set. According to the results, the AELS system gives the practices to the students, which helps to maximize the overall learning process. The network performance is continuously updated to reduce the deviation between the actual and predicted values. The system uses the bee colony approaches for updating the network performance values. The difference between the student learning and teaching process is computed using the error rate metrics. Then the obtained results are illustrated in Figure 6.

Figure 6 demonstrates the introduced Optimized Deep Learning Networks (ODNN) error rate on classroom and AELS-based learning processes. In the school, the students must listen to the teaching, and the students’ doubts are asked in the upcoming session. In the AELS system, students individually learn the concept and their mistakes and doubts are corrected automatically. The AELS learning process uses the expert’s knowledge, questionaries and templates while teaching the students. These templates are more practical for training the students at different learning levels. In addition, the COBW model generates the vectors for every word in the sentence. The created vectors are to understand and predict the following terms utilized in the sentence. This process is used to improve the overall learning efficiency. Here bee colony algorithm working process is utilized in the neural network updating process. The updating is done by using the probability value of the solution $\frac{f\,i\,t\,\left(x_{i}\right)}{\sum_{n=1}^{B\,N}f\,i\,t\,\left(x_{n}\right)}$. The computed solution is further evaluated using the mutual learning process. These procedures improve the overall network updating process. The continuous updating of network parameters minimizes the deviation between the learning inputs and output. In addition, the effectiveness of the AELS system is evaluated using a set of questions asked to the learners to analyze their learning ability. In this analysis, around ten students are selected, invited to answer, and their learning rate is illustrated in Table 2. Students are asked to perform the test by conducting the test and the efficiency is evaluated from their results. And gives ratings from 1 to 10.

**TABLE 2:** Learning efficiency analysis.

Questions	Classroom learning		ODNN-AELS system	
	Learning efficiency	Learning interest	Learning efficiency	Learning interest
1	4.8	5.9	8.93	9.34
2	4.92	5.19	9.09	9.54
3	6.7	5.83	9.34	9.62
4	5.34	6.2	9.76	9.48
5	6.1	6.45	9.84	9.84
6	6.4	6.23	9.76	9.7
7	5.98	6.02	9.45	9.8
8	6.02	6.12	9.74	9.45
9	6.34	6.2	9.74	9.87
10	6.3	6.28	8.97	9.03

**FIGURE 7:**
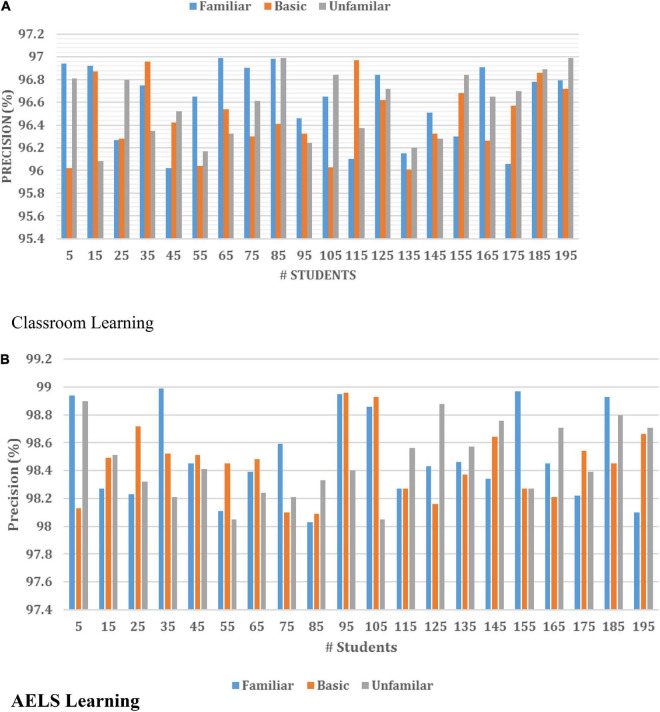
Precision analysis of **(A)** classroom and **(B)** AELS learning.

Table 2 illustrates the learning efficiency of the introduced HCI with the ODNN-based AELS system compared with the traditional learning process. The above results show that the students are interested in learning the English Language independently instead of using the classroom. During the analysis, 10 students are selected to examine their learning efficiency, in which a set of questions are asked to the students. From the investigation, the students are having 9.84% of learning rate while they are utilizing the AELS-based learning process. The AELS learning process can resolve the issues involved in the classroom and improve the overall learning rate. In addition, the system effectiveness is further evaluated using the precision and recall metrics. These metrics are utilized for how effectively the introduced ODNN-based AELS system satisfies the student’s requests. Then the obtained results are illustrated in Figure 7.

**FIGURE 8:**
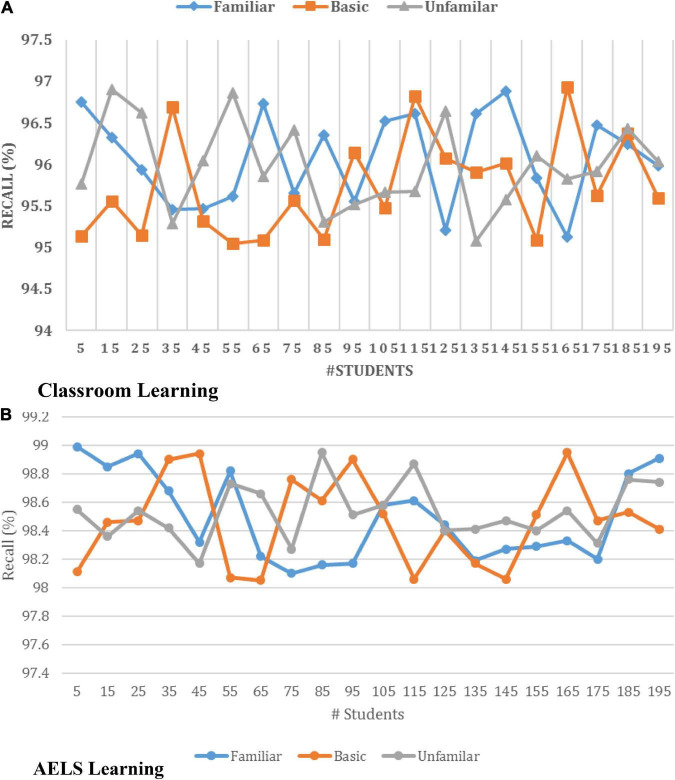
Recall Analysis of **(A)** classroom and **(B)** AELS learning.

Figure 7 illustrates that the introduced neural network effectively recognizes the user request-related words, vectors and patterns from the database. Here, the training set is created using extensive data to understand each query. The developed system can process the user request with any phases, such as familiar, basic and unfamiliar stages. The request-related vectors are retrieved and trained by applying the neural function, which helps predict the request-related response. In addition, the system corrects the student’s answer, and the right answers are given according to their learning process. The effective utilization of each learning function and activation process improves the overall learning rate. Then the system’s overall effectiveness is evaluated using the recall measure, and the results are illustrated in Figure 8.

Thus, Figure 8 clearly shows that the introduced ODNN approach-based AELS system ensures 98.51% accuracy while giving the training to the students. The effective utilization of the convolution layer, max polling function and fully convoluted layer in the COBW model helps achieve high accuracy while training the students. In addition, the network has an optimization algorithm-based network parameter updating procedure that minimizes the deviation between the actual content and student answer content. The minimum error rate indicates that the system effectively trains the students with maximum accuracy. Thus the automatic learning process improves the overall personal skills while learning independently. HCI simulation systems are used for college students’ spoken English instruction in this context from the standpoint of educational psychology. Using an interactive simulation system, a model mechanism for the interactive system, and a questionnaire about oral applied to learn education, and educational psychologists examine how an HCI simulation system affects college students’ oral English education strategy. The result is a more effective oral education strategy. This research focuses on the oral education of college students and may be extended to other fields to establish the groundwork for intelligent education.

## Conclusion

Thus the paper constructs the HCI with AELS by using Optimized Deep Learning Networks (ODNN). The analysis system uses the Japanese-English Bilingual Corpus information to create an effective independent learning set. The sentences in the English set are processed by NLP, removing irrelevant information. Then the word embeddings are derived from the words according to the optimized neural model. The neural model generates the patterns from the derived word that predicts the correct grammar and next word, which is used to train the student effectively. During the analysis, the bee colony optimization algorithm updates the network parameter, minimizing the deviation between the student and systematic answers. The minimum error rate indicates that the student independently learns English with maximum accuracy (98.51%). This work is able to deal with the four modules that is more relevant to the learning process; however, the method requires improvement while evaluating the student performance. In the future, the efficiency of the independent learning system will be further improved by utilizing the meta-heuristics optimization algorithm.

## Data availability statement

The original contributions presented in this study are included in the article/supplementary material, further inquiries can be directed to the corresponding author.

## Author contributions

XW: writing – original draft preparation. SS: editing data curation and supervision. Both authors contributed to the article and approved the submitted version.

## Footnotes

1
https://www.kaggle.com/team-ai/japaneseenglish-bilingual-corpus
